# Question answering systems for health professionals at the point of care—a systematic review

**DOI:** 10.1093/jamia/ocae015

**Published:** 2024-02-16

**Authors:** Gregory Kell, Angus Roberts, Serge Umansky, Linglong Qian, Davide Ferrari, Frank Soboczenski, Byron C Wallace, Nikhil Patel, Iain J Marshall

**Affiliations:** Department of Population Health Sciences, King’s College London, London, Greater London, SE1 1UL, United Kingdom; Department of Biostatistics and Health Informatics, King’s College London, London, Greater London, SE5 8AB, United Kingdom; Metadvice Ltd, London, Greater London, SW1Y 5JG, United Kingdom; Department of Biostatistics and Health Informatics, King’s College London, London, Greater London, SE5 8AB, United Kingdom; Department of Population Health Sciences, King’s College London, London, Greater London, SE1 1UL, United Kingdom; Department of Population Health Sciences, King’s College London, London, Greater London, SE1 1UL, United Kingdom; Khoury College of Computer Sciences, Northeastern University, Boston, MA 02115, United States; Department of Population Health Sciences, King’s College London, London, Greater London, SE1 1UL, United Kingdom; Department of Population Health Sciences, King’s College London, London, Greater London, SE1 1UL, United Kingdom

**Keywords:** clinical decision support, question answering, evidence-based medicine, natural language processing, artificial intelligence

## Abstract

**Objectives:**

Question answering (QA) systems have the potential to improve the quality of clinical care by providing health professionals with the latest and most relevant evidence. However, QA systems have not been widely adopted. This systematic review aims to characterize current medical QA systems, assess their suitability for healthcare, and identify areas of improvement.

**Materials and methods:**

We searched PubMed, IEEE Xplore, ACM Digital Library, ACL Anthology, and forward and backward citations on February 7, 2023. We included peer-reviewed journal and conference papers describing the design and evaluation of biomedical QA systems. Two reviewers screened titles, abstracts, and full-text articles. We conducted a narrative synthesis and risk of bias assessment for each study. We assessed the utility of biomedical QA systems.

**Results:**

We included 79 studies and identified themes, including question realism, answer reliability, answer utility, clinical specialism, systems, usability, and evaluation methods. Clinicians’ questions used to train and evaluate QA systems were restricted to certain sources, types and complexity levels. No system communicated confidence levels in the answers or sources. Many studies suffered from high risks of bias and applicability concerns. Only 8 studies completely satisfied any criterion for clinical utility, and only 7 reported user evaluations. Most systems were built with limited input from clinicians.

**Discussion:**

While machine learning methods have led to increased accuracy, most studies imperfectly reflected real-world healthcare information needs. Key research priorities include developing more realistic healthcare QA datasets and considering the reliability of answer sources, rather than merely focusing on accuracy.

## Background and significance

Despite a plethora of available evidence, health professionals find answers to only half of their questions, due to time constraints.^1–3^ This has motivated the development of online resources to answer clinicians’ questions based on the latest evidence. While scientifically rigorous information resources such as UpToDate, Cochrane, and PubMed exist, Google search remains the most popular resource used in practice.^4^ General-purpose search engines like Google offer ease-of-use, but rank results according to criteria that differ from evidence-based medicine principles of rigor, comprehensiveness, and reliability.^4^

To address these issues, there is burgeoning research into biomedical question answering (QA) systems.^5–13^ These could rival the accessibility and speed of Google or “curbside consultations” with colleagues, while providing answers based on reliable, up-to-date evidence. Moreover, Google is free to access, while services such as UpToDate charge for access and require manual updates. On the other hand, biomedical QA systems could be updated automatically. More recently, rapid advances in language modeling (particularly large language models [LLMs] such as GPT,^14^ and Galactica^15^) could allow healthcare professionals to request and receive natural language guidance summarizing evidence directly.

Many papers (eg, Refs. 5, 6, 8, 10, 16, 17) have described the development and evaluation of biomedical QA systems. However, the majority have not seen use in practice. We explored this problem previously,^18^ and argue that key reasons for non-uptake include answers which are not useful in real-life clinical practice (eg, yes/no, factoids, or answers not applicable to the locality or setting); systems that do not justify answers, communicate uncertainties, or resolve contradictions.^5^^,^^6^^,^^10^^,^^16^^,^^17^ Some existing papers have surveyed the literature on biomedical QA (eg, Refs. 19, 20) and found that few systems explain the reasoning for the returned answers, use all available domain knowledge, generate answers that reflect conflicting sources and are able to answer non-English questions.

Our contributions are to comprehensively characterize existing systems and their limitations, with the hope of identifying key issues whose resolution would allow for QA systems to be used in practice. We focus on complete QA systems as opposed to subcomponents.

## Methods

We conducted a systematic review and narrative synthesis of biomedical QA research, focusing on studies describing the development and evaluation of such systems. The protocol for this review is registered in PROSPERO (PROSPERO registration ID: CRD42021266053) and the Open Science Framework (OSF registration DOI: 10.17605/OSF.IO/4AM8D).

Studies were eligible if they were: (1) published in peer-reviewed conference proceedings and journals, (2) in English language, (3) described complete QA systems (ie, papers describing only subcomponent methods were excluded), evaluated the QA system (either based on a dataset of questions and answers, or a user study), (5) focused on biomedical QA for healthcare professionals. We excluded studies: (1) of QA systems for consumers/patients and (2) using modalities other than text, for example, vision. We searched PubMed, IEEE Xplore, ACM Digital Library, ACL Anthology, and forward and backward citations on February 7, 2023, using the following search strategy adapted for each database’s syntax:

(“question answering” OR “question-answering”) AND (clinic* OR medic* OR biomedic* OR health*)

Deduplicated titles and abstracts were double screened by G.K. (all) and D.F. and L.Q.A. (50% each). Disagreements were resolved via discussion, adjudicated by IJM. The same process was followed for full texts.

We used a structured data collection form which we refined after piloting (Appendix SA). We conducted a narrative synthesis following the steps recommended by Popay et al.^21^ Specifically, we conducted an initial synthesis by creating textual descriptions of each study and tabulating data on methods, datasets, evaluation methods, and findings, and creating conceptual maps. We assessed the robustness of findings via a risk of bias assessment, and by evaluating QA systems’ suitability for real-world use.

We evaluated the suitability of QA systems for use in practice, via criteria we developed previously and introduced in our position paper.^18^ This paper described how problems with transparency, trustworthiness, and provenance of health information contribute to the non-adoption of QA systems in real-world use. We proposed the following markers of high-quality QA systems. (1) Answers should come from reliable sources; (2) systems should provide guidance where possible; (3) answers should be relevant to the clinician’s setting; (4) sufficient rationale should accompany the answers; (5) conflicting evidence should be resolved appropriately; and (6) systems should consider and communicate uncertainties. We rated each system as completely, partially, or not meeting these criteria. We provide more detail regarding the application of these criteria in Appendix SB. Quality assessments were done in duplicate by G.K. (all papers), and L.Q. and D.F. (half of all papers each). Final assessments were decided through discussion and adjudicated by I.J.M.

In the absence of a directly relevant bias tool, we adapted PROBAST for use with QA studies.^22^ PROBAST evaluates study design, conduct, or analysis which can lead to biases in clinical predictive modeling studies. QA systems are like predictive models, but rather than predicting a diagnosis (based on some clinical criteria), they predict the best answer for a given question.

We adapted PROBAST to consider the quality of studies’ (1) questions (analogous to *population* in the original PROBAST), (2) input features (eg, bag-of-words, neural embeddings, etc., analogous to *predictors*), and (3) answers (analogous to *outcomes*). For each criterion, we assessed whether design problems led to *risk of bias*. We then assessed the studies for *applicability* concerns (ie, relevance of questions, models, and answers to general clinical practice). Risks of bias and applicability concerns were rated as high, low, or unclear for each paper. We provide the modified PROBAST in the Supplementary Materials; this may be useful to other researchers assessing QA systems. Other AI-focused tools (eg, APPRAISE-AI^23^) are rapidly becoming available; they cover similar aspects of bias to PROBAST.

We report our review according to the PRISMA^24^ and SwiM guidance.^25^ We provide raw data in the Supplementary Materials and present the final narrative synthesis below.

## Results

**Figure 1.**
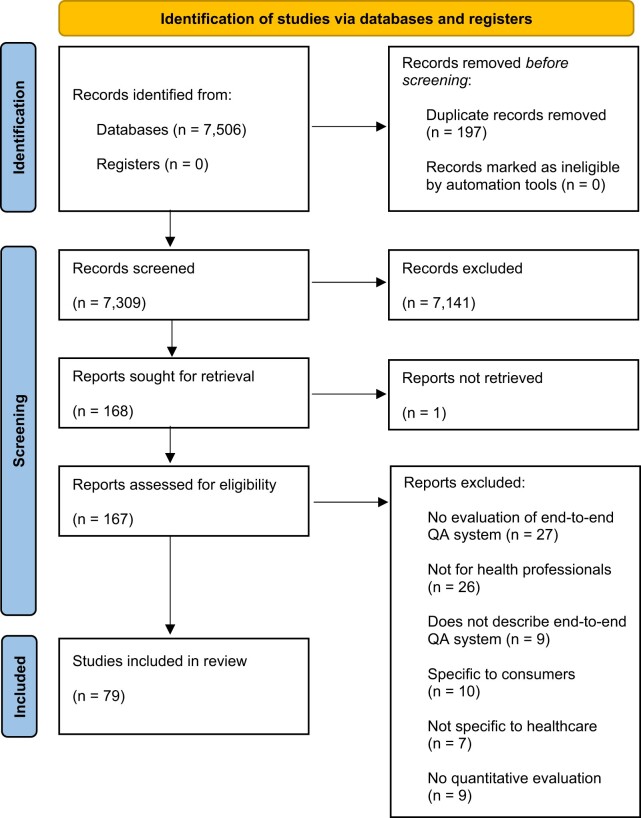
PRISMA flow diagram.

**Figure 2.**
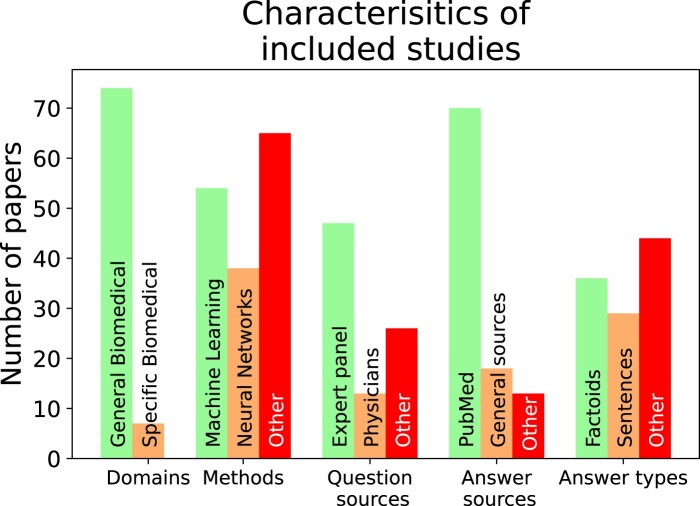
Number of papers with each category of domain, method, question, answer source, and answer type. The distinction was made between a major category and all the others, as one main category tended to dominate several smaller others. Table 1 contains more detail on the specifics of each paper.

The flow of studies, and reasons for inclusion/exclusion are shown in Figure 1. We included 79 of 7506 records identified in the searches in the final synthesis. Characteristics of included studies are described in Table 1 and Figure 2.

**Table 1.** Characteristics of included studies.

**Study**	Model/method	Evaluation question sources	Evaluation answer sources
Demner-Fushman et al (2006a)^31^	Semantic type classifier (UMLS, MeSH) PICO classifier Rule-based system Machine learning system	Physicians	PubMed
Demner-Fushman et al (2006b)^53^	Semantic-type classifier (UMLS) Clustering	Authors	PubMed
Lee et al (2006)^56^	Question classification Query term generation TF-IDF Document retrieval Lexico-syntactic patterns	Physicians	PubMed World Wide Web
Weiming et al (2007)^54^	Semantic-type classifier (UMLS) Semantic relation extraction BM25 TF-IDF Boolean search	Unclear	Medical documents
Demner-Fushman et al (2007)^11^	Semantic-type classifier (UMLS, MeSH) PICO classifier Rule-based system Machine learning system	Physicians	PubMed
Sondhi et al (2007)^69^	Semantic-type classifier (UMLS, ICD-9) Document ranking Clustering	Physicians	PubMed
Yu et al (2007) a^47^	User study of different systems	Physicians in practice	World Wide Web Online dictionaries PubMed
Yu et al (2007) b^17^	Naïve Bayes Lexico-syntactic patterns TF-IDF Information retrieval	Physicians in practice	World Wide Web PubMed
Makar et al (2008)^50^	Bayesian classifier Part of speech tagger Text extractor Summarizer	Physicians in practice	Wikipedia Google
Cao et al (2009)^60^	BM25 Term frequency Unique term frequency Longest common subsequence	Physicians	MEDLINE eMedicine documents Clinical guidelines PubMed Central Wikipedia
Gobeil et al (2009)^68^	MeSH descriptors Information retrieval Information extraction	Authors	PubMed
Pasche et al (2009a)^81^	Logical rules Information retrieval	Authors	PubMed
Pasche et al (2009b)^55^	Logical rules Information retrieval	Authors	PubMed
Xu et al (2009)^71^	Semantic-type classifier (UMLS) Question-type classifier Keyword extractor Passage retrieval Answer extraction	Unclear	Unclear
Olvera-Lobo et al (2010)^70^	START: open-domain QA system MedQA: restricted-domain QA system	Health website	START: Wikipedia Merriam-Webster Dictionary American Medical Association I MDB Yahoo Webopedia.com MedQA: MEDLINE Dictionary of Cancer Terms Wikipedia Google Dorland’s Illustrated Medical Dictionary Medline Plus Technical and Popular Medical Terms National Immunization Program Glossary
Tutos et al (2010)^28^	User study on different systems	Physicians	PubMed World Wide Web Brainboost
Cairns et al (2011)^12^	UMLS Rule-based algorithms Support vector machine	Physicians in practice	Medical wiki curated by approved physicians and doctoral-degreed biomedical students
Cao et al (2011)^5^	Semantic-type classifier (UMLS) Related questions extraction Information retrieval Information extraction Summarization	Unclear	Medical documents
Cruchet et al (2012)^72^	Semantic-type classifier (UMLS) Medical-term classifier Keyword-based retrieval	Physicians in practice	HONcode certified sites, for example, WebMD, Everyday Health, Drugs.com, and Healthline
Doucette et al (2012)^48^	Inference rules Semantic reasoner	Synthetic patient data	Synthetic patient data
Ni et al (2012)^29^	PICO classifier Rules-based system Template/pattern matching Information retrieval Machine learning system Answer candidate scoring	Medical health website	Medical health website
Ben Abacha and Zweigenbaum (2015)^6^	Semantic Web SPARQL Semantic graphs UMLS concepts UMLS semantic type Support vector machines Conditional random fields Rule-based methods	Physicians	PubMed
Gobeill et al (2015)^82^	Gene Ontology concepts Lazy pattern matching KNN BM25 Information retrieval	Authors	PubMed
Hristovski et al (2015)^57^	Semantic relation extraction (UMLS) Semantic relation retrieval	Authors	PubMed
Li et al (2015)^49^	Word2Vec Markov random field	Expert panel	PubMed
Tsatsaronis et al (2015)^52^	Comparison of different systems on the BioASQ dataset	Expert panel	PubMed
Vong et al (2015)^30^	PICO classifier Clustering	Authors	PubMed
Goodwin et al (2016)^8^	Knowledge graph Conditional random fields Bayesian inference	Unclear	Electronic health records PubMed
Yang et al (2016)^93^	Logistic Regression	Expert panel	PubMed
Brokos et al (2016)^103^	TF-IDF Word mover’s distance	Expert panel	PubMed
Krithara et al (2016)^94^	Comparison of different systems on the BioASQ dataset	Expert panel	PubMed
Sarrouti and El Alaoui (2017) a^102^	UMLS concepts BM25	Expert panel	PubMed
Sarrouti and El Alaoui (2017) b^95^	UMLS concepts BM25	Expert panel	PubMed
Jin et al (2017)^113^	Bag of words Term frequency Collection frequency Sequential dependence models Divergence from randomness models Multimodal strategies	Expert panel	PubMed
Neves et al (2017)^108^	Question processing (regular expressions, semantic types, named entities, keywords) Document/passage retrieval Answer extraction	Expert panel	PubMed
Wiese et al (2017a)^109^	RNN Domain adaptation	Expert panel	PubMed
Wiese et al (2017b)^110^	RNN Domain adaptation	Expert panel	PubMed
Nentidis et al (2017)^96^	Comparison of different systems on the BioASQ dataset	Expert panel	PubMed
Du et al (2018)^62^	GloVe LSTM Self-attention	Expert panel	PubMed
Eckert et al (2018)^98^	Semantic role labelling	Expert panel	PubMed
Papagiannopoulou et al (2016)^97^	Binary relevance models Linear SVMs Labeled LDA variant Prior LDA Fast XML HOMER-BR Multilabel ensemble	Expert panel	PubMed
Dimitriadis et al (2019)^73^	Word2Vec WordNet Custom textual features Logistic regression Support vector machine XGBoost	Expert panel	PubMed
Du et al (2019)^63^	GloVe LSTM Self-attention Cross-attention	Expert panel	PubMed
Jin Q et al (2019)^101^	BioBERT	Titles of papers	PubMed
Ozyurt et al (2019)^37^	GloVe BERT Inverse document frequency Relaxed word mover’s distance	Expert panel	PubMed
Jin ZX et al (2019)^61^	TF-IDF Noun extraction Part of speech tagger Semantic-type classifier (UMLS) Query expansion (MeSH) Markov random field Divergence from randomness Model ensemble	Expert panel	PubMed
Wasim et al (2019)^65^	Rules-based system Semantic-type classifier (UMLS) Logistic regression	Expert panel	PubMed
Oita et al (2020)^90^	Dynamic memory networks Bidirectional attention flow Transfer learning Biomedical named entity recognition Corroboration of semantic evidence	Expert panel	PubMed
Du et al (2020)^35^	BERT BiLSTM Self-attention	Expert panel	PubMed
Yan et al (2020)^112^	Binary classification RNNs Semi-supervised learning Recursive autoencoders	Expert panel	PubMed
Kaddari et al (2020)^58^	Survey of existing models	Expert panel	PubMed
Nishida et al (2020)^111^	BERT Domain adaptation Multitask learning	Expert panel Crowdworkers	PubMed Wikipedia
Omar et al (2020)^46^	Convolutional neural networks Attention Gated convolutions Gated attention	PubMed	PubMed
Ozyurt et al (2020a)^34^	GloVe BERT Inverse document frequency Relaxed word mover’s distance	Expert panel	PubMed
Ozyurt et al (2020b)^104^	ELECTRA	Expert panel	PubMed
Sarrouti et al (2020)^16^	Lexico-syntactic patterns Support vector machine Semantic-type classifier (UMLS) TF-IDF Semantic similarity-based retrieval BM25 Sentiment analysis	Expert panel	PubMed
Shin et al (2020)^99^	BioMegatron	Expert panel	PubMed
Wang et al (2020)^107^	Event extraction SciBERT	Authors	PubMed
Alzubi et al (2021)^32^	TF-IDF BERT	Authors	PubMed
Du et al (2021)^76^	QANet BERT GloVe Model weighting	Expert panel	PubMed
Nishida et al (2021)^100^	BERT fastText	Expert panel Crowdworkers	PubMed Wikipedia
Peng et al (2021)^39^	BERT BiLSTM Bagging	Expert panel	PubMed
Pergola et al (2021)^92^	BERT Masking strategies	Epidemiologists Medical doctors Medical students Expert panel	PubMed World Health Organization’s Covid-19 Database Preprint servers
Wu et al (2021)^27^	BERT Numerical encodings	Expert panel PubMed	PubMed
Xu et al (2021)^36^	BERT Syntactic and lexical features Feature fusion Transformer	Expert panel	PubMed
Bai et al (2022) a^79^	Dual encoder BioBERT	Expert panel	PubMed
Bai et al (2022) b^74^	Knowledge distillation Adversarial learning BioBERT	Expert panel	PubMed
Du et al (2022)^78^	QANet BERT GloVe Model weighting	Expert panel	PubMed
Kia et al (2022)^84^	Convolution neural network Attention	Authors	PubMed
Naseem et al (2022)^75^	ALBERT	Expert panel	PubMed
Pappas et al (2022)^105^	ALBERT-XL	Expert panel	PubMed
Raza et al (2022)^83^	BM25 MPNet	Expert panel	PubMed
Rakotoson et al (2022)^26^	BERT RoBERTa T5 Boolean classifier	Expert panel PubMed	PubMed
Wang et al (2022)^106^	Event extraction SciBERT Domain adaptation	Authors	PubMed
Weinzierl et al (2022)^77^	BERT BM25 Question generation Question entailment recognition	Expert panel	PubMed
Yoon et al (2022)^80^	BERT Sequence tagging BiLSTM-CRF	Expert panel	PubMed
Zhang et al (2022)^38^	BERT BM25	Expert panel	PubMed
Zhu et al (2022)^40^	BERT RoBERTa T5 XGBoost	PubMed	PubMed
Raza et al (2022)^85^	BM25 MPNet	Expert panel	PubMed

### Risk of bias, applicability, and utility

**Figure 3.**
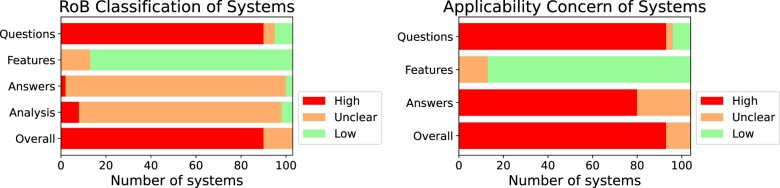
Number of papers achieving each risk of bias and applicability concern classification. Risk of bias refers to the risk of a divergence between the stated problem the paper tries to solve and the execution for reasons such as an unrealistic dataset or failing to split data for training and evaluation. Applicability refers to how applicable the system is to the review.

We summarize the risks of bias in Figure 3; individual study assessments are in the Supplementary Materials. 85% of systems had a high risk of bias overall; primarily driven by problems in the questions used to develop and evaluate the systems. Many studies used unrealistically simple questions or covered too few information needs for a general biomedical QA system. Most questions were hypothetical, and not generated by health professionals.

Most systems were at low risk of bias for defining and extracting machine learning (ML) features (eg, deciding on predictive features without reference to the reference answers). Most studies did not provide clear descriptions of answer data or evaluation methodology (eg, details about the source of answers) which led to unclear risk of bias assessments for most papers’ answers. Additionally, no answer was relevant to the biomedical QA domain. This led to high applicability concerns for most papers.

**Figure 4.**
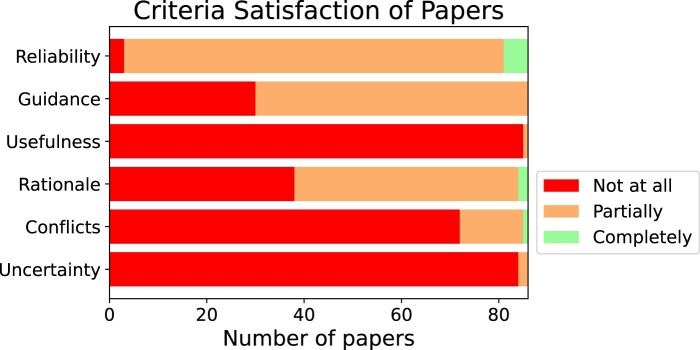
Number of papers achieving each satisfaction classification for each criterion.

We present utility scores in Figure 4. Few systems completely met any criterion. Two systems^26^^,^^27^ provided rationales (ie, justifications and sources) for their answers; 5 systems were judged to use reliable sources^11^^,^^28–31^; one system resolved conflicting information^26^ and one system communicated uncertainties.^26^ Very few systems provided contextually relevant answers (ie, locality-specific information, or specialty), while most systems provided clinical guidance at least partially (rather than basic science or less actionable information).

### Computational methods

**Figure 5.**
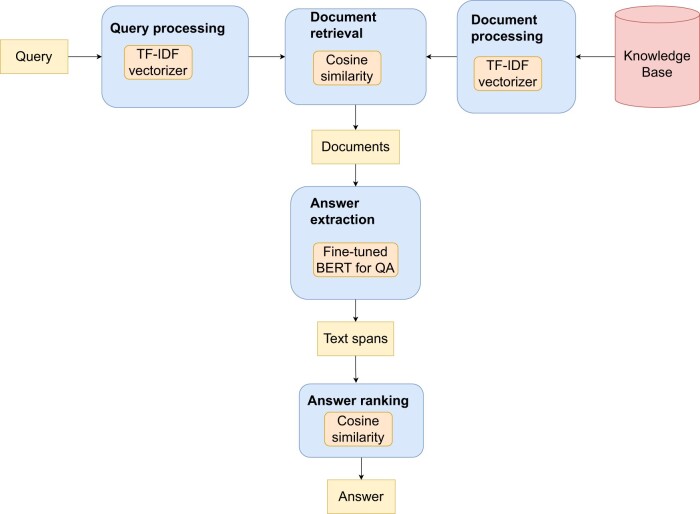
Typical QA architecture as used by Alzubi et al.^32^

Most QA systems used a knowledge base (ie, database of answers) that was created using documents from PubMed or other medical information sources (see Figure 5 for a typical example, from Alzubi et al^32^). Documents were either stored in structured form (knowledge graphs [KGs] or RDF triples) or as unstructured texts.

For a given user query, the system would retrieve the most relevant answer(s) from the knowledge base. KG-based (1 study), neural (24 studies) and modular systems (39 studies) were evaluated in the included studies (see Figure 2 and Appendix SC). KG-based systems accept natural language questions and convert them to KG-specific queries (eg, Cypher queries^33^) Modular systems comprise several distinct components (eg, question analysis, document retrieval, answer generation) designed separately and combined to form a QA system. Neural systems can be modular or monolithic.

All studies made use of datasets of questions with known answers. These datasets were used to train ML models (eg, document retrieval and answer extraction) and evaluate system performance. The topic focus of these datasets dictates the area(s) for which the QA can be successfully used; the quality of these datasets impacts both the accuracy of trained models and the reliability of the evaluations.

With regards to neural systems, 9 studies^32^^,^^34–40^ incorporated pretrained LLMs (eg, BERT,^41^ BioBERT,^42^ and GPT^43^) in their QA pipelines for text span extraction, sentence reranking and integrating sentiment information. These models were used to find potential answer text spans given questions and passages. Four studies^27^^,^^35^^,^^36^^,^^39^ found fine-tuning pretrained LLMs on biomedical data led to improvements in performance compared with only using only a general-domain LLM. No experiments were conducted on LLMs that were trained only on biomedical data.

**Figure 6.**
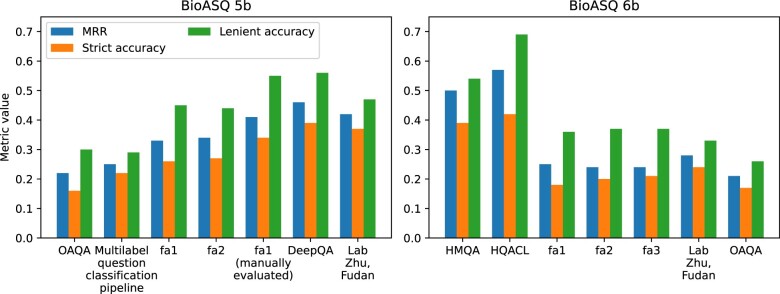
Results of the BioASQ 5b and 6b challenges for factoid-type answers.

Few studies used common datasets for training or evaluation. However, several of the included studies arose from the BioASQ 5b^44^ and 6b^45^ shared tasks, which aimed to answer 4 types of questions (yes/no, factoids, list, and summary questions) and had 2 phases: information retrieval and exact answer production. Three studies arising from BioASQ^53^^,^^54^^,^^65^ evaluated QA systems with a neural component, while 5 studies^52–54^^,^^57^^,^^65^ evaluated QA systems that relied only on rule-based or classical ML components (eg, support vector machines). The neural components encoded questions and passages with a recurrent neural network (RNN) that were then used to create intermediate representations before answers were generated with additional layers. Comparing results across the BioASQ studies suggests generally that QA systems employing ML components outperformed those that relied solely on rule-based components (see Figure 6 and Appendix SC).

Two papers included a numerical component in their QA pipelines. For example, one paper^27^ used numerical results (eg, odds ratios from clinical trial reports) to generate answers either to answer statistical questions (ie, “Do preoperative statins reduce atrial fibrillation after coronary artery bypass grafting?”). One study^27^ generated BERT-style embeddings using both textual and numerical encodings, leading to improved performance compared with using text alone.

### Topic areas

Fifty-three studies^5^^,^^8^^,^^11^^,^^16^^,^^17^^,^^26–31^^,34,^^35–40^^,^^46–80^ described QA systems covering a wide breadth of biomedical topics (Figure 2). These systems typically sourced answers from the unfiltered medical literature (eg, PubMed, covering both clinical practice guidelines and primary studies, including laboratory science and epidemiology). Eight studies examined specific specialties: one study focused on bacteriotherapy,^81^ 2 focused on genetics/genomics,^71^^,^^82^ and 5 on Covid-19.^32,77,^^83–85^ The genomics and Covid-19 systems were designed for specialists, while the bacteriotherapy system generated rules for managing antibiotic prescribing via a QA interface.

### Question datasets

Studies used several sources to generate question datasets (see Figure 2 and Appendix SD). We group these into questions collected from health professionals (either collected in the course of work or elicited as generate hypothetical questions; 14 studies), those generated by topic experts (13 studies), people without direct healthcare experience (eg, crowdworkers; 3 studies), and automatically/algorithmically derived (scraped from health websites, or generated from abstract titles; 2 studies). In 9 papers, questions were written by study authors.

Only 5^17^^,^^47^^,^^50^^,^^60^^,^^80^ studies used genuine questions posed by clinicians during consultations. Two studies^11^^,^^28^ used either simple or simplified questions. Examples of simple questions include “How to beat recurrent UTIs?”^28^ and “What is the best treatment for analgesic rebound headaches?”^11^ Questions the BioASQ challenge questions^52^ were created by an expert panel. BioASQ questions were restricted to yes/no, factoid and summary-type questions, and tended to have a highly technical focus. For example, the question “Which is the most common disease attributed to malfunction or absence of primary cilia?” could be answered with a factoid: “autosomal recessive polycystic kidney disease.” Alternatively, it could be answered with a summary (see Appendix SE for example). One study included definition questions created by the authors,^70^ while another^32^ included author-created factoid-style questions about a particular topic. Two studies^28^^,^^29^ utilized questions derived from health websites: one included questions generated by physicians^28^ and one^29^ used questions that were of unclear provenance.

While biomedical question sources enabled training of models, general domain QA datasets created using crowdworkers (eg, SQuAD^86^^,^^87^) were used to pretrain QA models in 3 studies.^35^^,^^62^^,^^63^ These pretrained models were then fine-tuned on biomedical QA datasets (eg, BioASQ^52^) Pretraining QA models on general crowdworker-created datasets prior to fine-tuning on biomedical datasets led to overall improvements in model performance in all 3 studies that explored this approach. In other words, pretraining on general-domain data led to an improvement in performance compared with training only on biomedical data.

### Reliability of answer sources

The answer sources used by the studies are summarized in Figure 2 and Appendix SG. Two studies^11^^,^^30^ found ranking biomedical articles by strength of evidence (based on publication types, source journals and study design) improved accuracy (eg, precision at 10 documents, mean average precision, mean reciprocal rank). None of the other studies accounted for differences in answer reliability within datasets (ie, information from major guidelines was treated equally to a letter to the editor).

Several studies included answers derived from health websites such as Trip Answers,^29^ WebMD,^70^ HON-certified websites,^72^ clinical guidelines and eMedicine documents (OSF registration DOI: 10.17605/OSF.IO/4AM8D). These answers were created by qualified physicians and underwent a review process. On the other hand, 3 studies^47^^,^^56^^,^^70^ explored systems that provided only term definitions from medical dictionaries. One study derived answers entirely from general domain sources,^28^ while another generated answers from a combination of medical and general sources. In the case of the latter, only the medical sources had a rigorous validation process.^70^ Two QA systems^29^^,^^72^ only derived the answers from health websites containing information that was vetted by the administrators. One study found that restricting the QA document collection based on trustworthiness increased the relevance of answers.^72^

### Detail of answers

Systems we reviewed varied in terms of what they produced as an “answer” (Figure 2 and Appendix SH). Answers consisting of only of one word (ie, cloze-style QA), factoids (a word or phrase, eg, aspirin 3g), list of factoids, or definitions were absolute in nature and therefore did not contain guidance (Appendix SH). On the other hand, contextual texts (eg, ideal answers^52^ and document summaries^16^) that accompanied absolute answers (eg, factoids) may have contained guidance. Similarly, biomedical articles accompanying answers consisting of medical concepts may have also included guidance, along with the sentences accompanying yes/no/unclear answers (see Appendix SH).

Several systems used a *clustered* approach to display answers. These systems grouped several candidate answers either by keyword or topics, eg, articles/sentences about heart conditions as one cluster. Clustered answers returned by the systems in 6 studies^5^^,^^30^^,^^53^^,^^54^^,^^60^^,^^69^ may contain guidance as the clusters are based around sentences, extracts of documents, or conclusions of abstracts. Other types of answers included abstracts and single/multiple sentences, documents/webpages, and URL-based answers (Appendix SH).

### Evaluation

Most studies (188) considered the accuracy of answers provided (see Table 2). Some assessed the degree to which the words in the answer match the reference, that is accuracy, precision, recall, F1 with respect to words (eg, ROUGE) or correct entire answers (eg, yes/no or factoids), numbers of answers/questions, exact matches. While ROUGE^88^ or BLEU^89^ may quantify the degree of similarity between candidate answers and the reference sentence, they are unable to account for, for example, negation or re-phrasings. Other systems were retrieval-based and so evaluated using the position of the correct answers in the returned list (ie, reciprocal rank, MAP, normalized discounted cumulative gain). Of the models that assessed accuracy/correctness, 31 used internal cross-validation, while 17 were evaluated on an independent dataset. Only 7 studies evaluated their design, system usability, or the relevance of the answer to the question as assessed by users. The most popular answer source was PubMed; most systems used a single source of answers.

**Table 2.** Grouping of papers according to accuracy metric.

Metric	Metric type	Papers	Number of papers
Accuracy	Accuracy/correctness	[16, 35, 36, 38–40, 46, 48, 50, 57, 58, 63, 65, 69, 73–75, 90, 92, 94–96, 98, 103–106, 109, 113]	29
Precision	Accuracy/correctness	[6, 11, 12, 16, 26, 29, 34, 40, 52, 54, 55, 57, 58, 65, 79, 80, 93–98, 103, 104, 107, 110–112]	28
Recall	Accuracy/correctness	[16, 26, 40, 52, 54, 58, 65, 70, 79–82, 93–96, 98, 103, 104, 107, 110–112]	24
Reciprocal rank	Accuracy/correctness	[6, 8, 12, 12, 16, 34, 35–39, 58, 62, 63, 65, 70, 71, 73, 74, 79, 94–96, 98, 100–107, 109]	32
F1	Accuracy/correctness	[16, 26, 29, 40, 52, 58, 62, 63, 65, 76, 78, 80, 83–85, 90, 93, 95, 96, 98, 100–105, 107–109, 111–113]	32
ROUGE	Accuracy/correctness	[16, 26, 31, 52, 90, 95, 96, 98, 100, 103]	10
Time taken to find answer	Usability	[5, 17, 26, 28, 47, 50	6
Likert score	Usability	5, 17, 28, 30, 47, 56, 60	7
Action frequency	Usability	17]	1
MAP	Accuracy/correctness	[52, 55, 61, 71, 91, 93, 99, 100, 104]	9
Numbers of queries/answers	Accuracy/correctness	[69–71, 100, 111]	5
Exact matches	Accuracy/correctness	[26, 32, 76, 78, 83–85, 107–109	10
Normalized discounted cumulative gain	Accuracy/correctness	77, 97]	2
AUC ROC	Accuracy/correctness	12	1

### Presentation and usability

Only 13 studies evaluated 7 systems that provided a user interface for user queries. These systems were MedQA,^17^^,^^28^^,^^47^^,^^70^^,^^114^ Omed,^48^ the system introduced in,^50^ EAGLi,^55^^,^^81^^,^^82^ AskHERMES,^5^^,^^30^^,^^60^ CQA-1.0,^30^ and CliniCluster.^30^ User interfaces are essential for assessing the performance of the systems with genuine users.

The only usability study^30^ assessed the effectiveness of a system that clustered answers to drug questions by I (intervention) and C (comparator) elements. The answers were tagged with P-O (patient-outcome) and I/C (intervention/comparator) elements (see Appendix SI for details). The participants agreed that the clustering of the answers helped them find answers more effectively, while more of the older participants found the P-O and I/C useful for finding relevant documents. Additionally, possessing prior knowledge about a given subject assisted with additional learning.

The ease of use of QA and IR systems was assessed in 3 studies.^5^^,^^48^^,^^17^ The systems evaluated included Google,^5^^,^^17^^,^^48^ MedQA,^17^^,^^48^ Onelook,^17^^,^^48^ PubMed,^17^^,^^48^ UpToDate,^5^ and AskHermes.^5^ Both Doucette et al^48^ and Yu et al^17^ rated Google as being the easiest to use, followed by MedQA, Onelook, and PubMed. On the other hand, Cao et al^5^ rated Google, UpToDate, and AskHermes equally in terms of ease of use.

None of the included systems presented any information about the certainty of answers; although nearly all systems used quantitative answer scoring to select the chosen answer. One study^60^ evaluated 2 approaches to presenting answers on the AskHermes system:^5^ passage-based (collection of several sentences) and sentence-based. The study found that passage-based approaches produced more relevant answers as rated by clinicians.

## Discussion

We systematically reviewed studies of the development and evaluation of biomedical QA systems, focusing on their merits and drawbacks, evaluation and analysis, and the overall state of biomedical QA. Most of the included studies had high overall risks of bias and applicability concerns. Few of the papers satisfied any utility criterion.^18^

Several studies highlight obstacles that should be overcome and measures that should be taken before deploying biomedical QA systems. For example, one general-domain QA user study^115^ found that users tended to prefer conventional search engines as they “felt less cognitive load” and “were more effective with it” than when they queried QA systems.

We note that commercial search engines are likely to benefit from comparatively vast development resources, and a focus on user experience. By contrast, the academic research we found tended to focus on the underlying computational methodology/models, with little attention to the user interface or experience—aspects which are likely highly influential in how QA systems are used.

Law et al^116^ found that presenting users with causal claims and scatterplots could lead users to accept unfounded claims. Nonetheless, warning users that “correlation is not causation” led to more cautious treatment of reasonable claims. Additionally, Schuff et al^118^ and Yang et al^117^ explored metrics for assessing the quality of the explanations: answer location score (LOCA) and the Fact-Removal score (FARM), F1 score, and exact matches.

More recently, there has been rapid development in LLMs, such as GPT,^14^ PaLM,^119^ and Med-PaLM,^120^ which are the current state of the art in natural language processing. There were 9 studies included that used LLMs, but they were used for text span extraction, sentence reranking and integrating sentiment information. A nascent application of LLMs is direct summarization of one or more sources. While LLMs can produce fluent answers to any given question,^121^ they are vulnerable to “hallucinating” plausible but fabricated information.^122–124^ This may be especially risky in healthcare due to the potentially life-threatening ramifications. One solution might be retrieval-augmented methods (where LLMS only use documents of known provenance). LLMs should be rigorously assessed before deployment in biomedical QA pipelines. This would ensure that the references provided by LLMs are genuine and that information is faithfully reproduced.

Barriers to adoption have been studied in detail in related technologies (eg, Clinical Decision Support Systems [CDSS]). Greenhalgh et al^125^^,^^126^ introduced the NASSS framework to characterize the complex reasons why technologies succeed (or fail) in practice; finding that aspects such as the dependability of the underlying technology and organizations’ readiness to adopt the new systems are critical. Similarly, Cimino and colleagues^127^ found that design issues (eg, time taken to answer each question, or the number of times a given link is clicked) were critical. We would argue that future QA research should take a broader view of evaluation if QA is to move from an academic computer science challenge to real-world benefit.

To our knowledge, this is the first systematic review of QA systems in healthcare. While other (non-systematic) reviews provide an overview of the biomedical QA field,^19^^,^^20^ we have evaluated existing systems and datasets for their utility in clinical practice. Furthermore, the inclusion of quantitative evaluations allowed for comparisons between different system types. Examination of questions, information sources, and answer types has allowed identification of factors that affect adherence to the criteria defined in.^18^

Most of the included studies were method papers describing systems that were built by computer scientists with limited input from clinicians. These systems were designed to perform well on benchmark datasets, such as BioASQ. While the studies were rigorous in their evaluation, they did consider how the systems could be used in practice. Future work should focus on translating biomedical QA research into practice.

One weakness is that we did not include purely qualitative evaluations. This might be a worthwhile SR to do in the future. We limited our search to published systems; therefore, this review would not have included any deployed systems which were not published; or systems described only in the “gray” literature (eg, pre-prints, PhD theses, etc.). We also did not search all the CDSS literature for pipelines incorporating QA systems. Deployment of such systems might not be described in the literature, as health providers may not have provided the results. Although we would expect most relevant papers to be published in English, there may have been pertinent non-English language papers that were missed.

### Implications for research

Studies to date have too often used datasets of factoids/multiple choice questions, which do not resemble real-life queries. There is a need for high-quality datasets derived from real clinical queries, and actionable high-quality clinical guidance.

Future research should move beyond maximizing accuracy of a model alone, and include aspects of transparency, answer certainty, and information provenance (is the reliability and source of answers understood by users?). These aspects will only become more important with the advent of LLMs, which tend to generate highly plausible and fluent answers, but are not always correct.

### Implications for practice

The performance of QA systems on biomedical tasks has increased over time, but the QA datasets were either unrepresentative of real-world information needs or were unrealistically simple. We recommend that practitioners exercise caution with any QA system which advertises accuracy only. Instead, systems should produce verifiable answers of known provenance, which make use of high-quality clinical guidelines and research.

## Conclusions

In this review, we reviewed the literature on QA systems for health professionals. Most studies assessed the accuracy of the systems on various datasets; only 13 evaluated the usability of the systems. Few studies explored practical usage of the systems, opting to compare them using QA benchmarks instead. Although none of the included studies described systems that completely satisfied our utility criteria, they discussed several characteristics that could be appropriate for future systems. These included, limiting the document collection to reliable sources, providing more verbose answers, clustering answers according to themes/categories and employing methodologies for numerical reasoning. Most of the papers used QA datasets which either were unrepresentative of real-world information needs, or were unrealistically simple (eg, factoids, yes/no). Thus, more realistic and complex datasets should be developed.

## Supplementary Material

ocae015_Supplementary_Data

## Data Availability

The data underlying this article are available in the article and in its online supplementary material.
