# Supplementary material for: Question answering systems for health professionals at the point of care—a systematic review
Source: J Am Med Inform Assoc. 2024 Feb 16;31(4):1009–24. doi: 10.1093/jamia/ocae015 (PMC10990539; doi:10.1093/jamia/ocae015)
Supplement: ocae015_Supplementary_Data [file ocae015_supplementary_data.zip › ocae015_Supplementary_Data/Appendix.docx]

Appendix

# SECTION A: DATA COLLECTION

The data collection form, piloted by GK, LQ and DF, was used to manually extract data from each included article. The variables extracted included publication year, author, journal title/conference name, article title, question answering domain, method and approach, source of questions for training and evaluation, source of answers for training and evaluation, form (i.e. factoid, definition, yes/no) of answers used for training and evaluation. We also evaluated the papers against the utility criteria outlined in [1], i.e. whether or not reliable sources of health information are used to derive the answers, whether or not the answers in the form of guidance, whether or not the answers useful In the context in which healthcare providers would be practising, whether or not there is sufficient “rationale” for the answers provided, whether or not the system resolve conflicting evidence appropriately, whether or not the system handle and communicate uncertainties adequately, size of the training set, size of the evaluation set, quantitative results based on the training data, and quantitative results based on the evaluation data.

# SECTION B: CRITERIA RATINGS

Table 1: Examples of texts that satisfy each criterion 'completely', 'partially' or 'not at all'.'

| **Domain** | **Examples that completely satisfy criterion** | **Example that partially satisfy criterion** | **Example that does not satisfy criterion at all** |
| --- | --- | --- | --- |
| **Reliable sources** | Sources that were verified and approved by medical professionals, e.g. HON-certified health websites.    The QA system contains a component that rates the reliability of answer sources when a mixture of sources is used. | Sources that have not been described/mentioned in the corresponding studies    Biomedical databases (e.g. PubMed) that contain a mixture of reliable (e.g. randomized control trials) and unreliable (e.g. opinion article) sources | Databases and search systems that contain and retrieve information that is predominantly non-medical in nature and has not been verified by medical professionals, e.g. Google. |
| **Answers in the form of guidance** | Answers that suggest guidelines that may be relevant, e.g. “No relevant local or national guidelines are available, but here is one from Wirral Community Teaching Hospital”.    Answers that provide conditional suggestions e.g. when recommending Nitrofurantoin: “If the estimated glomerular filtration rate (eGFR) ≥ 45 ml/minute then 100 mg modified-release twice a day (or if unavailable, 50 mg four times a day) for 3 days.” | Answers consisting of extracted text spans, sentences or paragraphs from a particular text that may be in the form of guidance. | Answers are in the form of factoids (e.g. 100g of aspirin) or single words.    Answers provide definitive but unverifiable instructions (e.g. “Prescribe X medicine for Y condition”). |
| **Useful in the context in which the provider is practising** | The answers are based on the location of the clinician, e.g. resources available to the hospital/clinic, antibiotic resistance. | Answers may account for one location-factor affecting the answer, e.g. antibiotic resistance, without considering any others. | The answers do not account for the situation in which the clinician is practising. |
| **Sufficient “rationale” for the answers provided** | The system supports a particular answer either with additional text, source links/references or both. | Answers consisting of extracted text spans, sentences or paragraphs from a particular text that may contain rationale. | The answer does not contain any explanation or references.  There is only a factoid, single word or phrase. |
| **Resolve conflicting evidence appropriately** | The system can identify conflicting evidence and communicate and communicate any conflicts to the clinicians, e.g. “3 systematic reviews were found, but their conclusions are contradictory”. | The system identifies conflicting evidence and chooses the most likely source without communicating the conflicts to the clinician.    The system recognizes the conflicting evidence and synthesises it accordingly into an answer without informing the clinician of the original sources. | The system assumes there is only one possible correct answer. |
| **Handle and communicate uncertainties adequately** | The system communicates any sources of uncertainty and abstains from providing explicit guidance where appropriate.  A good quality system would, for example, provide a caution if the answer came from low quality research evidence (a small or poor quality study). | The system identifies uncertain sources and excludes them from the answer but does not communicate their existence to the clinician. | The system is certain about every answer.    The system does not communicate uncertainties. |

# Section C: Types of systems

Table 2: Grouping of papers according to system type.

| **System type** | **Papers** |
| --- | --- |
| Knowledge graph | [2] |
| Neural | [3–29] |
| Modular | [2,4,5,11–13,17,19,22,23,27–60] |

Table 3: Results of the BioASQ 5b challenge, including source citation, system type, name and average metrics from batches 1-5.

|  | | | **Average results** | | |
| --- | --- | --- | --- | --- | --- |
| **Paper** | **Type of system** | **Name of system** | **MRR** | **Lenient Accuracy** | **Strict Accuracy** |
| [9] | Modular, neural | HMQA | **0.50** | - | - |
| [48] | Modular, classical ML | OAQA | 0.22 | 0.30 | 0.16 |
|  | Modular, classical ML | Proposed system | 0.25 | 0.29 | 0.22 |
| [12] | Modular, neural | fa1 | 0.33 | 0.45 | 0.26 |
|  | Modular, neural | fa2 | 0.34 | 0.44 | 0.27 |
|  | Modular, neural | fa1 (manually evaluated) | 0.41 | 0.55 | 0.34 |
|  | Modular, neural | Deep QA | 0.46 | **0.56** | **0.39** |
|  | Modular, rule-based | Lab Zhu, Fudan | 0.42 | 0.47 | 0.37 |

Table 4: Results of the BioASQ 6b challenge, including source citation, system type, name and average metrics from batches 1-5.

|  | | | **Average results** | | |
| --- | --- | --- | --- | --- | --- |
| **Paper** | **Type of system** | **Name of system** | **MRR** | **Lenient Accuracy** | **Strict Accuracy** |
| [10] | Modular, neural | HMQA | 0.50 | 0.54 | 0.39 |
|  | Modular, neural | HQACL | **0.57** | **0.69** | **0.42** |
| [12] | Modular, neural | fa1 | 0.25 | 0.36 | 0.18 |
|  | Modular, neural | fa2 | 0.24 | 0.37 | 0.20 |
|  | Modular, neural | fa3 | 0.24 | 0.37 | 0.21 |
|  | Modular,  rule-based | Lab Zhu, Fudan | 0.28 | 0.33 | 0.24 |
|  | Modular, classical ML | OAQA | 0.21 | 0.26 | 0.17 |

# SECTION D: SOURCES OF TRAINING/EVALUATION QUESTION DATA

Table 5: Grouping of papers according to question source.

| **Question source** | **Papers** | **Number of papers** |
| --- | --- | --- |
| Article titles and/or last sentences of abstracts | [3,20,25,61] | 4 |
| Physicians within clinical settings | [26,32,35,44,45,58] | 6 |
| Physicians who did not necessarily ask the questions in clinical settings | [30,31,36,39,41,50,51,53,55,59,62] | 11 |
| Expert panel | [3–5,7–13,15–24,26–29,34,37,46,48,52,60,62–78] | 46 |
| Health websites | [43,51] | 2 |
| Crowdworkers | [7,9,10,70,76,77] | 6 |
| Artificial conversations | [10] | 1 |
| Authors of the papers | [2,6,33,38,40,42,47,56,57,79,80] | 11 |
| Unclear sources | [14,39,54] | 3 |

With regards to sources of physicians’ questions that may not have been asked in clinical settings, four of the articles used the questions for user studies [30,41,45,55]. [31,36] had a high risk of bias and low applicability for the concern because although genuine physicians’ questions were used, either specifically simple ones were selected or compound questions were simplified. [53] had both a high risk of bias and applicability concern because the questions were created by one of the authors who had medical qualifications and the questions were designed to match specific answers. The questions may therefore not have been as complex as those asked in clinical settings. Similarly, [50] had a high risk of bias and applicability as the questions were created by novice physicians specifically for the study and thus may not be reflective of what clinicians would ask in practice.

The following studies used questions created by the authors according to a template: [33,38,40,42,47,56,57]. All the studies had high applicability concerns and a high risk of bias, apart from [40,56] which had a low risk of bias. This is because the target application was a tool for medical knowledge acquisition for clinical decision support. Even though the authors of [6,51] did not employ a specific template while creating the questions, they were still unrealistically simple. Hence, the studies were deemed to be of high risk of bias and had high applicability concerns. Furthermore, [51] focused only on definitional questions. As [47] is purely a user study, it had no associated risk of bias or applicability assessment.

# SECTION E: EXAMPLE OF SUMMARY ANSWER FOR BioASQ

“When ciliary function is perturbed, photoreceptors may die, kidney tubules develop cysts, limb digits multiply and brains form improperly. Malformation of primary cilia in the collecting ducts of kidney tubules is accompanied by development of autosomal recessive polycystic kidney disease”.

# SECTION F: SPECIALIZED QUESTION TOPICS

As the question and information sources of [50] were narrow, the risk of bias and applicability were high. All the drug-specific questions were also inapplicable to the review question for the same reason.

The questions used by [33,49,53] were deemed to be of high risk of bias because the research question of the paper exactly matched the review question. On the other hand, the systems developed in [40,56] addressed narrower research questions. Specifically, [40,56] aimed to create “machine-readable legacy knowledge rules” to generate guidelines for drug prescriptions.

# SECTION G: ANSWER SOURCES

Table 6: Grouping of papers according to answer source.

| **Answer source** | **Papers** | **Number of papers** |
| --- | --- | --- |
| PubMed/MEDLINE | [30,2,31,61,32,34,37,38,40,41,4,42,5,7,44,45,8,3,46,9,10,47,11,48–53,12,56,57,13–19,21–23,25,26,20,24,63–68,70,69,71–73,81,80,74–76,62,77,79,78,59,60,27–29] | 71 |
| Health websites | [43,51,55,58] | 4 |
| General QA websites | [36] | 1 |
| Online dictionaries | [32,41,51] | 3 |
| Preprints | [62] | 1 |
| Wikipedia | [7,9,10,30,35,70,76,77] | 8 |
| World Health Organisation | [62] | 1 |
| World wide web | [32,35,36,51,41,44] | 6 |
| Synthetic data | [33] | 1 |
| eMedicine documents | [30,45] | 2 |
| Clinical guidelines | [45] | 1 |
| Miscellaneous medical sources | [6,39,54] | 3 |

The limited control over the information contained in Wikipedia led to the answers derived using only Wikipedia [35] to be not at all reliable. Meanwhile, the answers that are derived from Wikipedia in tandem with other sources, e.g., biomedical databases and clinical notes, were partially reliable. The quality control over the information on Google is also limited, which is why answers derived only using Google are not at all reliable [36].

Additionally, general QA websites and miscellaneous medical sources were deemed to be partially reliable. This is because they were medical sources or, as in the case with the general QA websites, the answers to the questions were written by “topic experts”. The credentials of these “topic experts” are unknown. The synthetic data was not at all reliable as it may not be reflective of the real world.

# SECTION H: TYPES OF ANSWERS

Table 7: Grouping of papers according to answer type.

| **Answer type** | **Papers** | **Number of papers** |
| --- | --- | --- |
| One word | [61] | 1 |
| Medical concepts | [2,63,67] | 3 |
| Definitions | [4,35–37,44] | 5 |
| Yes/no/unclear answers | [5,20,25,27,29,33,37,52,59,63,64,66,71,73,75,81] | 16 |
| Clustered answers | [30,38,39,45,47,50] | 6 |
| Factoids | [5,8–10,12,13,15–18,23,24,37,40,48,49,52,56,57,59,62–64,68–80] | 36 |
| Lists of factoids | [5,9,26,48,63,64,66,68–71,73] | 12 |
| Abstracts | [53] | 1 |
| Single sentence | [14,19,22,25,43,51,81] | 7 |
| Paragraphs/several sentences | [4,6,11,20,30,31,36–38,41,42,44–47,51,58,64,68,71,73,81] | 22 |
| Documents/webpages | [36,50,58,63,65,67,68] | 7 |
| URLs | [35,36] | 2 |
| Snippets | [28,34,46,60,63,75] | 6 |
| Unclear | [51,55] | 2 |

These studies had answers that were judged to be not relevant to clinical practice in the RoB assessment and QA criteria. The approaches described in the studies included one word answers [61] and lists of factoids. In [61], a cloze-style approach to question answering was applied. Under this setting, a word would be removed from a sentence and the system should then predict the missing word.

From the definitions, only the systems in [32,35] and [36] (Onelook) consist of absolute definitions and do not satisfy any criteria. [4,37,44] provided sentences which may contain guidance. Hence, they partially satisfy the guidance criterion. [54] (paragraphs, documents and webpages), [37] (ideal answers) and [4] (extracted sentences) may contain rationale, while the systems described in the other papers do not.

For the yes/no/unclear answer types, some may contain guidance due to accompanying sentences or paragraphs [37,51] (START). Due to the absolute nature of the answer type, the other systems do not contain guidance. [37] partially satisfies the criterion due to the existence of “ideal” answers which may contain rationale. [20] completely satisfies the rationale and conflict resolution criterion, as the yes/no/balanced/neutral answers are accompanied by context and conflicts are resolved by majority votes. The systems outlined in the other papers do not offer any rationale.

The sentences of extracts of documents [30,38,39,45,50] used for the clustered answers may contain rationales. On the other hand, abstracts [53] and single/multiple sentences [43] may contain guidance or rationales. The snippet-based answers all contain partial guidance, but only [46] provides rationales. All the documents and webpages, i.e. [36] (Google, PubMed, MedQA) and [50], may contain guidance and rationales.

Out of the paragraph answer type (total of 16), all systems apart from 2 may contain guidance ([30,45] only provide definitions). In addition, half of the systems may provide rationales (except [16,17,30,33,45,57,63,64]).

URL-based answers were not in the form of guidance and did not offer rationales, as they are only accompanied by strict definitions. Example responses were not included in [51,52] (MedQA) and [55] nor was the format of the answers described. Therefore, it is impossible to determine the reliability of the answers, as well as whether they contain answers.

# SECTION I: USABILITY

The only usability study was conducted by [47] which assessed the usability of the CliniCluster system. The system answers only therapy questions and presents the users with a hierarchy of interventions which are clustered by the I (intervention) and C (comparator) elements in a collection of documents. When a particular cluster is selected, the user is shown a ranked list answers tagged with P-O (probability-outcome) and I/C (intervention/comparator) elements. The usability was evaluated using a survey which was answered by 20 medical professionals. The participants examined the 25 questions included in CliniCluster before answering the survey. Aside from questions about the usability, the survey collected demographic information about the participants such as age, gender, years of clinical experience and medical specialty. Additionally, the survey asked participants to rate how familiar and difficult the therapy topics were to them.

# SECTION J: REFERENCES

1. Kell G, Marshall I, Wallace B, Jaun A. What Would it Take to get Biomedical QA Systems into Practice? In: Proceedings of the 3rd Workshop on Machine Reading for Question Answering [Internet]. Punta Cana, Dominican Republic: Association for Computational Linguistics; 2021. p. 28–41. Available from: https://aclanthology.org/2021.mrqa-1.3

2. Goodwin TR, Harabagiu SM. Medical Question Answering for Clinical Decision Support. In: Proceedings of the . ACM International Conference on Information & Knowledge Management ACM International Conference on Information and Knowledge Management. 2016. p. 297–306.

3. Wu Y, Ting HF, Lam TW, Luo R. BioNumQA-BERT: Answering Biomedical Questions Using Numerical Facts with a Deep Language Representation Model. In: Proceedings of the 12th ACM Conference on Bioinformatics, Computational Biology, and Health Informatics [Internet]. New York, NY, USA: Association for Computing Machinery; 2021. (BCB ’21). Available from: https://doi.org/10.1145/3459930.3469557

4. Ozyurt IB, Bandrowski A, Grethe JS. Bio-AnswerFinder: a system to find answers to questions from biomedical texts. Database : the journal of biological databases and curation. 2020;2020.

5. Kaddari Z, Mellah Y, Berrich J, Bouchentouf T, Belkasmi MG. Biomedical Question Answering: A Survey of Methods and Datasets. 2020 Fourth International Conference On Intelligent Computing in Data Sciences (ICDS). 2020;1–8.

6. Alzubi JA, Jain R, Singh A, Parwekar P, Gupta M. COBERT: COVID-19 Question Answering System Using BERT. Arabian journal for science and engineering. 2021;1–11.

7. Du Y, Pei B, Zhao X, Ji J. Deep scaled dot-product attention based domain adaptation model for biomedical question answering. Methods (San Diego, Calif). 2020;173:69–74.

8. Xu G, Rong W, Wang Y, Ouyang Y, Xiong Z. External features enriched model for biomedical question answering. BMC Bioinformatics. 2021 May 26;22(1):272.

9. Du Y, Pei B, Zhao X, Ji J. Hierarchical Multi-layer Transfer Learning Model for Biomedical Question Answering. 2018 IEEE International Conference on Bioinformatics and Biomedicine (BIBM). 2018;362–7.

10. Du Y, Guo W, Zhao Y. Hierarchical Question-Aware Context Learning with Augmented Data for Biomedical Question Answering. 2019 IEEE International Conference on Bioinformatics and Biomedicine (BIBM). 2019;370–5.

11. I. B. Ozyurt, J. Grethe. Iterative Document Retrieval via Deep Learning Approaches for Biomedical Question Answering. In: 2019 15th International Conference on eScience (eScience). 2019. p. 533–8.

12. Dimitriadis D, Tsoumakas G. Word embeddings and external resources for answer processing in biomedical factoid question answering. Journal of biomedical informatics. 2019;92:103118.

13. Raza S, Schwartz B, Ondrusek N. A Question-Answering System on COVID-19 Scientific Literature. In: 2022 IEEE 46th Annual Computers, Software, and Applications Conference (COMPSAC). 2022. p. 1331–6.

14. Kia MA, Garifullina A, Kern M, Chamberlain J, Jameel S. Adaptable Closed-Domain Question Answering Using Contextualized CNN-Attention Models and Question Expansion. IEEE Access. 2022;10:45080–92.

15. Bai J, Yin C, Zhang J, Wang Y, Dong Y, Rong W, et al. Adversarial Knowledge Distillation Based Biomedical Factoid Question Answering. IEEE/ACM Trans Comput Biol Bioinform. 2022 Mar 22;PP.

16. Naseem U, Dunn AG, Khushi M, Kim J. Benchmarking for biomedical natural language processing tasks with a domain specific ALBERT. BMC Bioinformatics. 2022 Apr 21;23(1):144.

17. Raza S, Schwartz B, Rosella LC. CoQUAD: a COVID-19 question answering dataset system, facilitating research, benchmarking, and practice. BMC Bioinformatics. 2022 Jun 2;23(1):210.

18. Du Y, Yan J, Zhao Y, Lu Y, Jin X. Dual Model Weighting Strategy and Data Augmentation in Biomedical Question Answering. In: 2021 IEEE International Conference on Bioinformatics and Biomedicine (BIBM). 2021. p. 659–62.

19. Weinzierl MA, Harabagiu SM. Epidemic Question Answering: question generation and entailment for Answer Nugget discovery. J Am Med Inform Assoc. 2023 Jan 18;30(2):329–39.

20. Rakotoson L, Letaillieur C, Massip S, Laleye FAA. Extractive-Boolean Question Answering for Scientific Fact Checking. In: Proceedings of the 1st International Workshop on Multimedia AI against Disinformation [Internet]. New York, NY, USA: Association for Computing Machinery; 2022. p. 27–34. (MAD ’22). Available from: https://doi.org/10.1145/3512732.3533580

21. Du Y, Yan J, Lu Y, Zhao Y, Jin X. Improving Biomedical Question Answering by Data Augmentation and Model Weighting. IEEE/ACM Trans Comput Biol Bioinform. 2022 Apr 29;PP.

22. Bai J, Yin C, Wu Z, Zhang J, Wang Y, Jia G, et al. Improving Biomedical ReQA With Consistent NLI-Transfer and Post-Whitening. IEEE/ACM Transactions on Computational Biology and Bioinformatics. 2022;1–12.

23. Zhang X, Jia Y, Zhang Z, Kang Q, Zhang Y, Jia H. Improving End-to-End Biomedical Question Answering System. In: Proceedings of the 8th International Conference on Computing and Artificial Intelligence [Internet]. New York, NY, USA: Association for Computing Machinery; 2022. p. 274–9. (ICCAI ’22). Available from: https://doi.org/10.1145/3532213.3532254

24. Peng K, Yin C, Rong W, Lin C, Zhou D, Xiong Z. Named Entity Aware Transfer Learning for Biomedical Factoid Question Answering. IEEE/ACM Trans Comput Biol Bioinform. 2021 May 11;PP.

25. Zhu X, Chen Y, Gu Y, Xiao Z. SentiMedQAer: A Transfer Learning-Based Sentiment-Aware Model for Biomedical Question Answering. Front Neurorobot. 2022;16:773329.

26. Yoon W, Jackson R, Lagerberg A, Kang J. Sequence tagging for biomedical extractive question answering. Bioinformatics. 2022 Aug 2;38(15):3794–801.

27. Oita M, Vani K, Oezdemir-Zaech F. Semantically Corroborating Neural Attention for Biomedical Question Answering. In: Cellier P, Driessens K, editors. Machine Learning and Knowledge Discovery in Databases. Cham: Springer International Publishing; 2020. p. 670–85.

28. Yan Y, Zhang BW, Li XF, Liu Z. List-wise learning to rank biomedical question-answer pairs with deep ranking recursive autoencoders. PLoS One. 2020;15(11):e0242061.

29. Arabzadeh N, Bagheri E. A self-supervised language model selection strategy for biomedical question answering. Journal of Biomedical Informatics. 2023 Sep;146:104486.

30. Cao Y, Liu F, Simpson P, Antieau L, Bennett A, Cimino JJ, et al. AskHERMES: An online question answering system for complex clinical questions. Journal of biomedical informatics. 2011;44(2):277–88.

31. Demner-Fushman D, Lin J. Answering Clinical Questions with Knowledge-Based and Statistical Techniques. Computational Linguistics. 2007;33:63–103.

32. Yu H, Kaufman D. A cognitive evaluation of four online search engines for answering definitional questions posed by physicians. Pac Symp Biocomput. 2007;328–39.

33. Doucette JA, Khan A, Cohen R. A Comparative Evaluation of an Ontological Medical Decision Support System (OMeD) for Critical Environments. In: Proceedings of the 2nd ACM SIGHIT International Health Informatics Symposium [Internet]. New York, NY, USA: Association for Computing Machinery; 2012. p. 703–8. (IHI ’12). Available from: https://doi.org/10.1145/2110363.2110444

34. Li Y, Yin X, Zhang B, Liu T, Zhang Z, Hao H. A Generic Framework for Biomedical Snippet Retrieval. 2015 3rd International Conference on Artificial Intelligence, Modelling and Simulation (AIMS). 2015;91–5.

35. Makar R, Kouta M, Badr A. A Service Oriented Architecture for Biomedical Question Answering System. 2008 IEEE Congress on Services Part II (services-2 2008). 2008;73–80.

36. Tutos A, Mollá D. A Study on the Use of Search Engines for Answering Clinical Questions. In: Proceedings of the Fourth Australasian Workshop on Health Informatics and Knowledge Management - Volume 108. AUS: Australian Computer Society, Inc.; 2010. p. 61–8. (HIKM ’10).

37. Tsatsaronis G, Balikas G, Malakasiotis P, Partalas I, Zschunke M, Alvers MR, et al. An overview of the BIOASQ large-scale biomedical semantic indexing and question answering competition. BMC bioinformatics. 2015;16:138.

38. Demner-Fushman D, Lin J. Answer Extraction, Semantic Clustering, and Extractive Summarization for Clinical Question Answering. In: Proceedings of the 21st International Conference on Computational Linguistics and the 44th Annual Meeting of the Association for Computational Linguistics [Internet]. USA: Association for Computational Linguistics; 2006. p. 841–8. (ACL-44). Available from: https://doi.org/10.3115/1220175.1220281

39. W. Weiming, D. Hu, M. Feng, L. Wenyin. Automatic Clinical Question Answering Based on UMLS Relations. In 2007. p. 495–8.

40. Pasche E, Teodoro D, Gobeill J, Ruch P, Lovis C. Automatic medical knowledge acquisition using question-answering. Studies in health technology and informatics. 2009;150:569–73.

41. Lee M, Cimino J, Zhu H, Sable C, Shanker V, Ely J, et al. Beyond Information Retrieval—Medical Question Answering. AMIA Annu Symp Proc. 2006 Feb;469–73.

42. Hristovski D, Dinevski D, Kastrin A, Rindflesch TC. Biomedical question answering using semantic relations. BMC bioinformatics. 2015;16(1):6.

43. Ni Y, Zhu H, Cai P, Zhang L, Qui Z, Cao F. CliniQA : highly reliable clinical question answering system. Studies in health technology and informatics. 2012;180:215–9.

44. Yu H, Lee M, Kaufman D, Ely J, Osheroff JA, Hripcsak G, et al. Development, implementation, and a cognitive evaluation of a definitional question answering system for physicians. Journal of biomedical informatics. 2007;40(3):236–51.

45. Cao YG, Ely J, Antieau L, Yu H. Evaluation of the Clinical Question Answering Presentation. In: Proceedings of the Workshop on Current Trends in Biomedical Natural Language Processing. USA: Association for Computational Linguistics; 2009. p. 171–8. (BioNLP ’09).

46. Jin ZX, Zhang BW, Fang F, Zhang LL, Yin XC. Health assistant: answering your questions anytime from biomedical literature. Bioinformatics (Oxford, England). 2019;35(20):4129–39.

47. Vong W, Then PHH. Information seeking features of a PICO-based medical question-answering system. 2015 9th International Conference on IT in Asia (CITA). 2015;1–7.

48. M. Wasim, W. Mahmood, M. N. Asim, M. U. Khan. Multi-Label Question Classification for Factoid and List Type Questions in Biomedical Question Answering. IEEE Access. 2019;7:3882–96.

49. Gobeill J, Patsche E, Theodoro D, Veuthey A, Lovis C, Ruch P. Question answering for biology and medicine. 2009 9th International Conference on Information Technology and Applications in Biomedicine. 2009;1–5.

50. Sondhi P, Raj P, Kumar VV, Mittal A. Question processing and clustering in INDOC: a biomedical question answering system. EURASIP J Bioinform Syst Biol. 2007;2007(1):28576.

51. Olvera-Lobo MD, Gutiérrez-Artacho J. Question-answering systems as efficient sources of terminological information: an evaluation. Health Info Libr J. 2010 Dec;27(4):268–76.

52. Sarrouti M, Ouatik El Alaoui S. SemBioNLQA: A semantic biomedical question answering system for retrieving exact and ideal answers to natural language questions. Artif Intell Med. 2020 Jan;102:101767.

53. Demner-Fushman D, Lin J. Situated Question Answering in the Clinical Domain: Selecting the Best Drug Treatment for Diseases. In: Proceedings of the Workshop on Task-Focused Summarization and Question Answering. USA: Association for Computational Linguistics; 2006. p. 24–31. (SumQA ’06).

54. B. Xu, H. Lin, B. Liu. Study on question answering system for biomedical domain. In: 2009 IEEE International Conference on Granular Computing. 2009. p. 626–9.

55. Cruchet S, Boyer C, van der Plas L. Trustworthiness and relevance in web-based clinical question answering. Studies in health technology and informatics. 2012;180:863–7.

56. Pasche E, Teodoro D, Gobeill J, Ruch P, Lovis C. QA-driven guidelines generation for bacteriotherapy. AMIA Annu Symp Proc. 2009 Nov 14;2009:509–13.

57. Gobeill J, Gaudinat A, Pasche E, Vishnyakova D, Gaudet P, Bairoch A, et al. Deep Question Answering for protein annotation. Database : the journal of biological databases and curation. 2015;2015.

58. Cairns B, Nielsen RD, Masanz JJ, Martin JH, Palmer M, Ward W, et al. The MiPACQ clinical question answering system. AMIA . Annual Symposium proceedings AMIA Symposium. 2011;2011:171–80.

59. Ben Abacha A, Zweigenbaum P. MEANS: A medical question-answering system combining NLP techniques and semantic Web technologies. Information Processing & Management. 2015 Sep;51(5):570–94.

60. Sarrouti M, Ouatik El Alaoui S. A passage retrieval method based on probabilistic information retrieval model and UMLS concepts in biomedical question answering. J Biomed Inform. 2017 Apr;68:96–103.

61. Omar R, El-Makky N, Torki M. A Character Aware Gated Convolution Model for Cloze-style Medical Machine Comprehension. 2020 IEEE/ACS 17th International Conference on Computer Systems and Applications (AICCSA). 2020;1–7.

62. Pergola G, Kochkina E, Gui L, Liakata M, He Y. Boosting Low-Resource Biomedical QA via Entity-Aware Masking Strategies. In: Proceedings of the 16th Conference of the European Chapter of the Association for Computational Linguistics: Main Volume [Internet]. Association for Computational Linguistics; 2021. Available from: https://doi.org/10.18653%2Fv1%2F2021.eacl-main.169

63. Yang Z, Zhou Y, Nyberg E. Learning to Answer Biomedical Questions: OAQA at BioASQ 4B. In: Proceedings of the Fourth BioASQ workshop [Internet]. Association for Computational Linguistics; 2016. Available from: https://doi.org/10.18653%2Fv1%2Fw16-3104

64. Krithara A, Nentidis A, Paliouras G, Kakadiaris I. Results of the 4th edition of BioASQ Challenge. In: Proceedings of the Fourth BioASQ workshop [Internet]. Association for Computational Linguistics; 2016. Available from: https://doi.org/10.18653%2Fv1%2Fw16-3101

65. Brokos GI, Malakasiotis P, Androutsopoulos I. Using Centroids of Word Embeddings and Word Mover’s Distance for Biomedical Document Retrieval in Question Answering. In: Proceedings of the 15th Workshop on Biomedical Natural Language Processing [Internet]. Association for Computational Linguistics; 2016. Available from: https://doi.org/10.18653%2Fv1%2Fw16-2915

66. Sarrouti M, Alaoui SOE. A Biomedical Question Answering System in BioASQ 2017. In: BioNLP 2017 [Internet]. Association for Computational Linguistics; 2017. Available from: https://doi.org/10.18653%2Fv1%2Fw17-2337

67. Jin ZX, Zhang BW, Fang F, Zhang LL, Yin XC. A Multi-strategy Query Processing Approach for Biomedical Question Answering: USTB_PRIR at BioASQ 2017 Task 5B. In: BioNLP 2017 [Internet]. Association for Computational Linguistics; 2017. Available from: https://doi.org/10.18653%2Fv1%2Fw17-2348

68. Neves M, Eckert F, Folkerts H, Uflacker M. Assessing the performance of Olelo, a real-time biomedical question answering application - BioNLP 2017. BioNLP 2017 [Internet]. 2017; Available from: https://doi.org/10.18653%2Fv1%2Fw17-2344

69. Wiese G, Weissenborn D, Neves M. Neural Domain Adaptation for Biomedical Question Answering - Proceedings of the 21st Conference on Computational Natural Language Learning (CoNLL 2017). Proceedings of the 21st Conference on Computational Natural Language Learning (CoNLL 2017) [Internet]. 2017; Available from: https://doi.org/10.18653%2Fv1%2Fk17-1029

70. Wiese G, Weissenborn D, Neves M. Neural Question Answering at BioASQ 5B - BioNLP 2017. BioNLP 2017 [Internet]. 2017; Available from: https://doi.org/10.18653%2Fv1%2Fw17-2309

71. Nentidis A, Bougiatiotis K, Krithara A, Paliouras G, Kakadiaris I. Results of the fifth edition of the BioASQ Challenge - BioNLP 2017. BioNLP 2017 [Internet]. 2017; Available from: https://doi.org/10.18653%2Fv1%2Fw17-2306

72. Papagiannopoulou E, Papanikolaou Y, Dimitriadis D, Lagopoulos S, Tsoumakas G, Laliotis M, et al. Large-Scale Semantic Indexing and Question Answering in Biomedicine. In: Proceedings of the Fourth BioASQ workshop [Internet]. Association for Computational Linguistics; 2016. Available from: https://doi.org/10.18653%2Fv1%2Fw16-3107

73. Eckert F, Neves M. Semantic role labeling tools for biomedical question answering: a study of selected tools on the BioASQ datasets. In: Proceedings of the 6th BioASQ Workshop A challenge on large-scale biomedical semantic indexing and question answering [Internet]. Association for Computational Linguistics; 2018. Available from: https://doi.org/10.18653%2Fv1%2Fw18-5302

74. Shin HC, Zhang Y, Bakhturina E, Puri R, Patwary M, Shoeybi M, et al. BioMegatron: Larger Biomedical Domain Language Model. In: Proceedings of the 2020 Conference on Empirical Methods in Natural Language Processing (EMNLP) [Internet]. Association for Computational Linguistics; 2020. Available from: https://doi.org/10.18653%2Fv1%2F2020.emnlp-main.379

75. Ozyurt IB. On the effectiveness of small, discriminatively pre-trained language representation models for biomedical text mining. In: Proceedings of the First Workshop on Scholarly Document Processing [Internet]. Association for Computational Linguistics; 2020. Available from: https://doi.org/10.18653%2Fv1%2F2020.sdp-1.12

76. Nishida K, Nishida K, Saito I, Asano H, Tomita J. Unsupervised Domain Adaptation of Language Models for Reading Comprehension - Proceedings of the Twelfth Language Resources and Evaluation Conference. Calzolari N, Béchet F, Blache P, Choukri K, Cieri C, Declerck T, et al., editors. Proceedings of the Twelfth Language Resources and Evaluation Conference. 2020 May;5392–9.

77. Nishida K, Nishida K, Yoshida S. Task-adaptive Pre-training of Language Models with Word Embedding Regularization. In: Findings of the Association for Computational Linguistics: ACL-IJCNLP 2021 [Internet]. Association for Computational Linguistics; 2021. Available from: https://doi.org/10.18653%2Fv1%2F2021.findings-acl.398

78. Pappas D, Malakasiotis P, Androutsopoulos I. Data Augmentation for Biomedical Factoid Question Answering. In: Proceedings of the 21st Workshop on Biomedical Language Processing [Internet]. Association for Computational Linguistics; 2022. Available from: https://doi.org/10.18653%2Fv1%2F2022.bionlp-1.6

79. Wang XD, Leser U, Weber L. BEEDS: Large-Scale Biomedical Event Extraction using Distant Supervision and Question Answering. In: Proceedings of the 21st Workshop on Biomedical Language Processing [Internet]. Association for Computational Linguistics; 2022. Available from: https://doi.org/10.18653%2Fv1%2F2022.bionlp-1.28

80. Wang XD, Weber L, Leser U. Biomedical Event Extraction as Multi-turn Question Answering. In: Proceedings of the 11th International Workshop on Health Text Mining and Information Analysis [Internet]. Association for Computational Linguistics; 2020. Available from: https://doi.org/10.18653%2Fv1%2F2020.louhi-1.10

81. Jin Q, Dhingra B, Liu Z, Cohen W, Lu X. PubMedQA: A Dataset for Biomedical Research Question Answering. 2019. 2567 p.
