# Supplementary material for: Question answering systems for health professionals at the point of care—a systematic review
Source: J Am Med Inform Assoc. 2024 Feb 16;31(4):1009–24. doi: 10.1093/jamia/ocae015 (PMC10990539; doi:10.1093/jamia/ocae015)
Supplement: ocae015_Supplementary_Data [file ocae015_supplementary_data.zip › ocae015_Supplementary_Data/RoB Assessments.zip!/RoB Assessments/Medical Question Answering for Clinical Decision Support.docx]

**PROBAST**

(Prediction model study Risk Of Bias Assessment Tool)

Published in Annals of Internal Medicine (freely available):

1. [PROBAST: A Tool to Assess the Risk of Bias and Applicability of Prediction Model Studies](https://annals.org/aim/fullarticle/2719961/probast-tool-assess-risk-bias-applicability-prediction-model-studies)
2. [PROBAST: A Tool to Assess Risk of Bias and Applicability of Prediction Model Studies: Explanation and Elaboration](https://annals.org/aim/fullarticle/2719962/probast-tool-assess-risk-bias-applicability-prediction-model-studies-explanation)

| **Step** | **Task** | **When to complete** |
| --- | --- | --- |
| **1** | Specify your systematic review question(s) | Once per systematic review |
| **2** | Classify the type of prediction model evaluation | Once for each model of interest in each publication being assessed, for each relevant outcome |
| **3** | Assess risk of bias and applicability | Once for each development and validation of each distinct prediction model in a publication |
| **4** | Overall judgment | Once for each development and validation of each distinct prediction model in a publication |

**What does PROBAST assess?**

PROBAST assesses both the *risk of bias* and *concerns regarding applicability* of a study that evaluates (develops, validates or updates) a multivariable diagnostic or prognostic prediction model. It is designed to assess primary studies included in a systematic review.

*Bias* occurs if systematic flaws or limitations in the design, conduct or analysis of a primary study distort the results. For the purpose of prediction modelling studies, we have defined *risk of bias* to occur when shortcomings in the study design, conduct or analysis lead to systematically distorted estimates of a model’s predictive performance or to an inadequate model to address the research question. Model predictive performance is typically evaluated using calibration, discrimination and sometimes classification measures, and these are likely inaccurately estimated in studies with high risk of bias. *Applicability* refers to the extent to which the prediction model from the primary study matches your systematic review question, for example in terms of the participants, predictors or outcome of interest.

A primary study may include the development and/or validation or update of more than one prediction model. A PROBAST assessment should be completed for each distinct model that is developed, validated or updated (extended) for making individualised predictions. Where a publication assesses multiple prediction models, only complete a PROBAST assessment for those models that meet the inclusion criteria for your systematic review. Please note that subsequent use of the term “model” includes derivatives of models, such as simplified risk scores, nomograms, or recalibrations of models.

PROBAST is not designed for all multivariable diagnostic or prognostic studies. For example, studies using multivariable models to identify predictors associated with an outcome but not attempting to develop a model for making individualised predictions are not covered by PROBAST.

PROBAST includes four steps.

If this is your first time using PROBAST, we strongly recommend reading the detailed explanation and elaboration (E&E, see link above) paper and to check the examples on www.probast.org

# Step 1: Specify your systematic review question

State your systematic review question to facilitate the assessment of the applicability of the evaluated models to your question. *The following table should be completed once per systematic review.*

| **Criteria** | **Specify your systematic review question** |
| --- | --- |
| *Intended use of model:* | *Question answering for clinicians, clinical decision support* |
| ***Participants*** *including selection criteria and setting:* | *Questions relevant to general clinical*  *practice* |
| ***Predictors*** *(used in prediction modelling), including types of predictors (e.g. history, clinical examination, biochemical markers, imaging tests), time of measurement, specific measurement issues (e.g., any requirements/ prohibitions for specialized equipment):* | Class of outcome, class of measure, text of the question, context/type of consultation where question was asked, metadata.  Features extracted from questions and information sources:  i.e. word/sentence/paragraph/document embeddings, UMLS concepts, semantic features etc. |
| *Outcome to be predicted:* | *Answers to clinicians’ questions arising during consultations with patients in primary care, which could be the top documents/text snippets containing the answer.* |

# Step 2: Classify the type of prediction model evaluation

Use the following table to classify the evaluation as model development, model validation or model update, or combination. Different signalling questions apply for different types of prediction model evaluation. If the evaluation does not fit one of these classifications then PROBAST should not be used.

| **Classify the evaluation based on its aim** | | |  |
| --- | --- | --- | --- |
| **Type of prediction study** | **PROBAST boxes to complete** | **Tick as appropriate** | **Definition for type of prediction model study** |
| Development only | Development |  | Prediction model development without external validation. These studies may include internal validation methods, such as bootstrapping and cross-validation techniques. |
| Development and validation | Development and validation |  | Prediction model development combined with external validation in other participants in the same article. |
| Validation only | Validation | Y | External validation of existing (previously developed) model in other participants. |

| *This table should be completed once for each publication being assessed and for each relevant outcome in your review.* | |
| --- | --- |
| **Publication reference** | Goodwin 2016 |
| **Models of interest** | Large-scale QA system based on EHRs and biomedical articles |
| **Outcome of interest** | Accuracy |

# Step 3: Assess risk of bias and applicability

PROBAST is structured as four key domains. Each domain is judged for risk of bias (low, high or unclear) and includes signalling questions to help make judgements. Signalling questions are rated as yes (Y), probably yes (PY), probably no (PN), no (N) or no information (NI). All signalling questions are phrased so that “yes” indicates absence of bias. Any signalling question rated as “no” or “probably no” flags the potential for bias; you will need to use your judgement to determine whether the domain should be rated as “high”, “low” or “unclear” risk of bias. The guidance document contains further instructions and examples on rating signalling questions and risk of bias for each domain.

The first three domains are also rated for concerns regarding applicability (low/ high/ unclear) to your review question defined above.

*Complete all domains separately for each evaluation of a distinct model. Shaded boxes indicate where signalling questions do not apply and should not be answered.*

| **DOMAIN 1: Participants** | | | |
| --- | --- | --- | --- |
| **A. Risk of Bias** | | | |
| *Describe the sources of data and criteria for question selection:*    How was the data (questions, information sources and example answers) for the QA systems collected?  In order to better enable access to the scientific literature in the clinical setting, research is necessary to evaluate information retrieval methods that connect clinical notes with the published literature. The TREC Clinical Decision Support (CDS) track simulates the information retrieval requirements of such systems to encourage the creation of tools and resources necessary for their implementation. In 2014 and 2015, the CDS tracks used simulated patient cases presented as if they were typical case reports used in medical education. However, in an actual electronic health record (EHR), patient notes are written in a much different manner, notably with terse language and heavy use of abbreviations and clinical jargon. To address the challenge specific to EHR notes, the 2016 CDS track used de-identified notes for actual patients. This enabled partici- pants to experiment with a realistic topic/query and develop methods to handle the challenging nature of clinical text. For a given EHR note, participants were challenged with retrieving full-text biomedical articles relevant for answering questions related to one of three generic clinical information needs: Diagnosis (i.e., “What is this patient’s diagnosis?”), Test (“What diagnostic test is appropriate for this patient?”), and Treatment (“What treatment is appropriate for this patient?”). Retrieved articles were judged relevant if they provided information of the specified type useful for the given case. The assessment was performed by physicians with training in biomedical informatics using a 3-point scale: relevant, partially relevant, not relevant.  The full-text article collection used for the track was drawn from the open access subset of PubMed Central (PMC)1, as in 2014 and 2015. However, an updated snapshot was used since the original snapshot was from January 2014. PMC is an online digital database of freely available full-text biomedical literature. The full text of each article is represented as an NXML file (XML encoded using the U.S. National Library of Medicine (NLM) Journal Archiving and Interchange Tag Library)2. Images and other supplemental ma- terials were also available. Each article in the collection is identified by a unique number (PMCID) that was used for run submissions. The PMCID of an article is specified by the <article-id> element within its NXML file. To make processing the document collection easier for the participants, each article file in the collection was renamed according to the article’s PMCID. For example, an article with PMCID 3148967 was renamed 3148967.nxml. The articles were available for download in 4 file bundles containing all 1.25 million articles in the snapshot.  The topics for the track were nursing admission notes obtained from the MIMIC-III database (Johnson et al., 2016). The MIMIC team gave the track special permission to make 30 notes publicly available without the need for a Data Use Agreement (DUA). MIMIC-III notes have already been automatically de-identified, but to ensure maximal privacy protection, manual de-identification was performed as well. Further, only notes from deceased patients were used.  MIMIC records were obtained from Boston-area Intensive Care Units (ICU). Since the patients are un- dergoing critical care, their medical problems are necessarily less diverse than the topics from previous years. However, there is still a wide diversity of ICU patients. To help ensure a diversity of cases selected as topics, the notes were automatically clustered using K-means with 30 clusters according to the patient’s associated ICD-9 codes (billing codes that roughly represent the patient’s problems). Topic creators were given a cluster with a selection of up to 10 admission notes. They chose one of the notes in the cluster to be the topic, discarding the rest. Where necessary, topic creators removed parts of the note to hide details of the actual diagnosis, test, or treatment performed. Topic creators also trimmed the notes to reduce overall length when necessary, removing sections that largely were not manually entered (but added automatically from structured fields within the EHR). Specifically, topic creators kept the History of Present Illness (HPI) section, which corresponds to the “case report” used in previous tracks. Once a clinical note was selected, manually de-identified, and edited where appropriate, the topic creators then constructed a case description and summary similar to the 2014 and 2015 tasks. | | | |
|  | | Dev | Val |
| 1.1 Was the difficulty of questions realistic? | |  | N |
| 1.2 Do the questions span a realistic spread of topics? | |  | PY |
| 1.3 Were the questions used to develop and evaluate the question answering systems representative of those that would be asked in the target application? | |  | PY |
| **Risk of bias introduced by selection of participants** | **RISK:**  *(low/ high/ unclear)* |  | **high** |
| *Rationale of bias rating:*  The target application is biomedical QA for clinicians. The questions were unrealistically simplistic to be used by clinicians. | | | |
| **B. Applicability** | | | |
| *Describe included participants, setting and dates:*  Were the questions developed using real questions asked by clinicians in primary care? No. They are derived from topics used in TREC CDS.  Were the questions used to develop and evaluate the question answering systems representative of questions that would be asked by clinicians in general clinical practice? No. They were too simplistic. | | | |
| **Concern that the included participants and setting do not match CONCERN:**  high  **the review question** *(low/ high/ unclear)* | | | |
| *Rationale of applicability rating:*  The questions were not genuine questions asked by physicians in clinical settings and they were too simplistic. | | | |

| **DOMAIN 2: Predictors** | | |  |
| --- | --- | --- | --- |
| **A. Risk of Bias** | | |  |
| *List and describe predictors included in the final model, e.g. definition and timing of assessment:*    i.e. features extracted from query and information sources?  The query was represented by a topic t and the articles and Electronic Health Records were represented by a knowledge graph of medical concepts. | | |  |
|  | | Dev | Val |
| 2.1 Were predictors defined and assessed in a similar way for all participants? | |  | Y |
| 2.2 Were predictor assessments made without knowledge of outcome data? | |  | Y |
| 2.3 Are all predictors available at the time the model is intended to be used? | |  | PY |
| **Risk of bias introduced by predictors or their assessment** | **RISK:**  *(low/ high/ unclear)* |  | **low** |
| *Rationale of bias rating:*  All features were extracted consistently for queries, EHRs and articles. | | |  |
| **B. Applicability** | | |  |
| Concern that the definition, assessment or timing of predictors in the model do not match the review question  If contextual data not used as a predictor, hospital ward or metadata used to predict answer. System which used unrealistic predictor | **CONCERN:**  *(low/ high/ unclear)* |  | **low** |
| *Rationale of applicability rating:*  Feature attributes match review question and no additional contextual data is required. | | |  |

| **DOMAIN 3: Outcome** | | | |
| --- | --- | --- | --- |
| **A. Risk of Bias** | | | |
| *Describe the outcome, how it was defined and determined, and the time interval between predictor assessment and outcome determination:*    What was the form and content of the answers?  Medical concepts consisting of one of more words, accompanied by ranked list of articles containing answer. | | | |
|  | | Dev | Val |
| 3.1 Was the outcome determined appropriately? | |  | PY |
| 3.2 Was a pre-specified or standard outcome definition used? | |  | Y |
| 3.3 Were predictors excluded from the outcome definition? | |  | Y |
| 3.4 Was the outcome defined and determined in a similar way for all participants? | |  | PY |
| 3.5 Was the outcome determined without knowledge of predictor information? | |  | Y |
| 3.6 Were the answers derived from reliable sources? | |  | NI |
| **Risk of bias introduced by the outcome or its determination** | **RISK:**  *(low/ high/ unclear)* |  | **Unclear** |
| *Rationale of bias rating:*  Unclear whether the answers were derived from reliable sources. | | | |
| **B. Applicability** | | | |
| *At what time point was the outcome determined:*    Were the answers selected relevant to the domain of interest. Yes.  Describe the quality of the assignment of answers to questions (i.e. clinical/information specialist?). Topic creators were given a cluster with a selection of up to 10 admission notes. They chose one of the notes in the cluster to be the topic, discarding the rest. Where necessary, topic creators removed parts of the note to hide details of the actual diagnosis, test, or treatment performed. Topic creators also trimmed the notes to reduce overall length when necessary, removing sections that largely were not manually entered (but added automatically from structured fields within the EHR). Specifically, topic creators kept the History of Present Illness (HPI) section, which corresponds to the “case report” used in previous tracks. Once a clinical note was selected, manually de-identified, and edited where appropriate, the topic creators then constructed a case descrip- tion and summary similar to the 2014 and 2015 tasks.  Were the answers likely to produce useful guidance in practice? Unclear whether the accompanying articles contained guidance.  *If a composite outcome was used, describe the relative frequency/distribution of each contributing outcome:* | | | |
| **Concern that the outcome, its definition, timing or determination do not match the review question** | **CONCERN:**  *(low/ high/ unclear)* |  | **unclear** |
| *Rationale of applicability rating:*  Unclear whether the articles contained guidance. | | | |

| **DOMAIN 4: Analysis** | | | |
| --- | --- | --- | --- |
| **Risk of Bias** | | | |
| *Describe numbers of participants, number of candidate predictors, outcome events and events per candidate predictor:*    Number of questions in the train and test sets.  Test set – 37,807 | | | |
| *Describe how the model was developed (for example in regards to modelling technique (e.g. survival or logistic modelling), predictor selection, and risk group definition):*    Architecture of system  In this paper, we present a novel Q/A from KB method used for answering the medical questions evaluated in TREC-CDS. First, instead of relying on an existing large KB, we automatically generated a very large medical knowledge graph from a publicly available collection of EMRs. As reported in [23], the medical case descriptions from the TREC-CDS topics were generated by consulting the EMRs from MIMIC-II [17]. Consequently, we used all the publicly available EMRs provided by MIMIC-III (a more recent superset of the EMRs in MIMIC-II) to automatically generate a very large probabilistic knowledge graph designed to encode knowledge acquired from medical practice. Second, instead of retrieving answers directly from the KB, the answers were obtained through probabilistic inference methods. Third, instead of identifying the answers from relevant PubMed articles, we used the answers inferred from the knowledge graph to select and rank the PubMed articles which contain them. In this way, we replaced the architecture of the typical system that was evaluated in TREC-CDS, illustrated in Figure 2(a) with a new architecture, illustrated in Figure 2(b). The new architecture identifies the ranked list of answers to the questions as well as the ranked list of scientific articles that contain them. While current state-of-the-art systems that were evaluated in TREC-CDS first processed the topics and then used query expansion methods to enhance the relevance of the retrieved scientific articles from PubMed, as reported in [23] and illustrated in Figure 2(a), we relied on a knowledge graph encoding the clinical picture and therapy of a large population of patients documented in the MIMIC III EMR database. The automatic generation of the knowledge graph involved: (1) medical language processing to identify medical concepts representing signs/symptoms, diagnoses, tests and treatments, as well as their assertions; (2) the cohesive properties of the medical narratives from the EMRs; and (3) a factorized Markov network representation of the medical knowledge. As illustrated in Figure 2(b), this probabilistic knowledge graph was used for inferring the answer of the TREC-CDS topics, which were processed to discern the medical concepts and their assertions in the same format as the nodes from the knowledge graph.   | | | |
| *Describe whether and how the model was validated, either internally (e.g. bootstrapping, cross validation, random split sample) or externally (e.g. temporal validation, geographical validation, different setting, different type of participants):*    Externally – no data driven methods were used to develop QA system. | | | |
| *Describe the performance measures of the model, e.g. (re)calibration, discrimination, (re)classification, net benefit, and whether they were adjusted for optimism:*    F1, ROUGE, Precision@k etc.  (1) the inferred Average Precision (iAP), wherein retrieved articles were randomly sampled and the Average Precision was calculated as in [37]; (2) the inferred Normalized Discounted Cumulative Gain (iNDCG), wherein retrieved articles were randomly sampled and the NDCG was calculated as per [38]; (3) the R-Precision, which measures the precision of the highest R-retrieved documents, where R is the total number of relevant documents for the topic; and (4) the Precision of the first ten documents retrieved (P@10). | | | |
| *Describe any participants who were excluded from the analysis:*    *N/A* | | | |
| *Describe missing data on predictors and outcomes as well as methods used for missing data:*  N/A | | | |
|  | | Dev | Val |
| 4.1 Were there a reasonable number of participants with the outcome? | |  | Y |
| 4.2 Were continuous and categorical predictors handled appropriately? | |  | PY |
| 4.3 Were all enrolled participants included in the analysis? | |  | Y |
| 4.4 Were participants with missing data handled appropriately? | |  | NI |
| 4.5 Was selection of predictors based on univariable analysis avoided? | |  |  |
| 4.6 Were complexities in the data (e.g. censoring, competing risks, sampling of controls) accounted for appropriately? | |  | NI |
| 4.7 Were relevant model performance measures evaluated appropriately? | |  | Y |
| 4.8 Were model overfitting and optimism in model performance accounted for? | |  |  |
| 4.9 Do predictors and their assigned weights in the final model correspond to the results from multivariable analysis? | |  |  |
| **Risk of bias introduced by the analysis** | **RISK:**  *(low/ high/ unclear)* |  | **low** |
| *Rationale of bias rating:*  Unclear how missing data and complexities in the data were handled. | | | |

# Step 4: Overall assessment

| **Reaching an overall judgement about risk of bias of the prediction model evaluation** | |
| --- | --- |
| **Low risk of bias** | If all domains were rated low risk of bias.  If a prediction model was developed without any external validation, and it was rated as low risk of bias for all domains, consider downgrading to **high risk of bias**. Such a model can only be considered as low risk of bias, if the development was based on a very large data set and included some form of internal validation. |
| **High risk of bias** | If at least one domain is judged to be at **high risk of bias**. |
| **Unclear risk of bias** | If an unclear risk of bias was noted in at least one domain and it was low risk for all other domains. |

| **Reaching an overall judgement about applicability of the prediction model evaluation** | |
| --- | --- |
| **Low concerns regarding applicability** | If low concerns regarding applicability for all domains, the prediction model evaluation is judged to have **low concerns regarding applicability**. |
| **High concerns regarding applicability** | If high concerns regarding applicability for at least one domain, the prediction model evaluation is judged to have **high concerns regarding applicability**. |
| **Unclear concerns regarding applicability** | If unclear concerns (but no “high concern”) regarding applicability for at least one domain, the prediction model evaluation is judged to have **unclear concerns regarding applicability** overall. |

Use the following tables to reach overall judgements about risk of bias and concerns regarding applicability of the prediction model evaluation (development and/or validation) across all assessed domains. *Complete for each evaluation of a distinct model.*

| **Overall judgement about risk of bias and applicability of the prediction model evaluation** | | |
| --- | --- | --- |
| **Overall judgement of risk of bias** | **RISK:**  *(low/ high/ unclear)* | **high** |
| *Summary of sources of potential bias:*  The target application is biomedical QA for clinicians. The questions were unrealistically simplistic to be used by clinicians.  Unclear how missing data and complexities in the data were handled. | | |
| **Overall judgement of applicability** | **CONCERN:**  *(low/ high/ unclear)* | **high** |
| *Summary of applicability concerns:*  The questions were not genuine questions asked by physicians in clinical settings and they were too simplistic. | | |
